# Global, regional, and national disease burden of lymphoma and leukemia attributable to high body mass index: from 1990 to 2021

**DOI:** 10.3389/fnut.2025.1592443

**Published:** 2025-07-24

**Authors:** Li Zhou, Dongyong Shan, Yingzhe Zhang, Jiwei Li, RenFang Deng

**Affiliations:** ^1^Department of Pulmonary and Critical Care Medicine, The Second Xiangya Hospital, Central South University, Changsha, China; ^2^Research Unit of Respiratory Disease, Central South University, Changsha, China; ^3^Diagnosis and Treatment Center of Respiratory Disease, Central South University, Changsha, China; ^4^Clinical Medical Research Center for Pulmonary and Critical Care Medicine in Hunan Province, Changsha, China; ^5^Department of Oncology, The Second Xiangya Hospital, Central South University, Changsha, China; ^6^Department of Oncology, The Second Hospital of Zhuzhou City, Zhuzhou, China

**Keywords:** hematological malignancies, leukemia, lymphoma, GBD, BMI

## Abstract

**Background:**

Lymphoma and leukemia were the most common hematological malignancies worldwide. Metabolic factors, such as high body mass index, are potential risk factors for various cancers. This study aimed to analyze the disease burden and the incidence trend of non-Hodgkin and acute leukemia attributable to high BMI around the world from 1990 to 2021.

**Methods:**

Using the data released by the Global Burden of Disease study 2021 (GBD 2021), we analyze the disease burden of non-Hodgkin lymphoma and acute leukemia attributable to high BMI from 1990 to 2021 via morbidity, death, disability-adjusted life years (DALY), and predict the obesity-related death trend in for the next 20 years (2022–2046).

**Results:**

The death cases of non-Hodgkin lymphoma, acute myeloid leukemia and acute lymphoid leukemia attributable to BMI in 2021 were 12,796, 11,947 and 4,116 respectively, with an increase of 153,9, 169.9, and 92.6% compared with 1990. The age-standardized mortality rate (ASMR) in 2021 attributable to BMI grew from 0.13/100,00, 0.11/100,000 and 0.05/100,000 in 1990 to 0.15/100,00, 0.14/100,00 and 0.05/100,000 in 2021. In 2021, the number of deaths cases and DALYs case were generally higher in male population and older population. The prediction of the neural network model showed that the incidence and death of the disease would remain high and rise in the next 25 years.

**Conclusion:**

High BMI has become a risk factor for leukemia and lymphoma and threatened public health globally. We should pay more attention to the role of metabolic factors and made more proactive and effective strategies.

## Introduction

Lymphoma and leukemia were the most common hematological malignancies worldwide. According to the latest cancer statistics released in 2024, there are approximately 89,190 new cases of lymphoma 62,770 new cases of leukemia every year in United States, resulting in 21,050 and 23,670 cancer related deaths, respectively (1). The causes of lymphoma and leukemia remain complicated, although various genetic factors such as somatic driver mutation have been implicated (2).

In addition to genetic factors, such as c-myc rearrangement (3, 4), some non-genetic factors, including virus infection, high BMI (Body mass index) status, smoking, and alcohol consumption, are demonstrated to be major risk factors for the development of hematological malignancies (5–7). Previous studies have demonstrated that immune function can be modified by obesity and high BMI was associated with around 20 cancers (8, 9). The above results indicated that high BMI status might be a potential hazard for lymphoma and leukemia.

In this study, original data from the Global Burden of Disease study 2021 (GBD 2021) were employed to inspect the disease burden of lymphoma and leukemia attributable to BMI in time, space, and population characteristics, focusing on non-Hodgkin lymphoma and acute leukemia, and to predict the disease trends in the next 20-year cycle, which may provide fundamental basis for the policy formulation and allocation of health resources.

## Materials and methods

### Data source

The datasets utilized in this study were derived from the Global Burden of Disease (GBD) 2021 study and accessed through the Global Health Data Exchange (GHDx) platform.^1^ These datasets provide comprehensive information on global disease burden and associated risk factors. Non-Hodgkin lymphoma and acute leukemia were classified according to the International Classification of Diseases, 10th Revision (ICD-10) codes (C82–C86.6, C96–C97.9 and C56, respectively). Acute myeloid leukemia (AML) incidence was further validated using ICD-9 codes (92.0–C92.02, C92.3–C92.62, C93.0–C93.02, C94.0–C94.02, C94.2–C94.22, C94.4–C94.5) and ICD-10 codes (205.0–205.02, 205.2–205.32, 206.0–206.02, 207.0–207.02, 207.2–207.82).

To assess the disease burden, several metrics were employed, including cancer incidence, mortality, disability-adjusted life years (DALYs), age-standardized mortality rate (ASMR), age-standardized DALY rate (ASDR), and estimated annual percentage changes (EAPCs). DALYs were calculated as the sum of years of life lost (YLL) and years lived with disability (YLD). The methodology used in the GBD study has been extensively documented in prior publications. Within the GBD risk factor hierarchy and its accompanying exposure definitions, a high BMI (>25 kg/m^2^) is defined relative to the theoretical minimum risk exposure level, which is set at a BMI of 20–25 kg/m^2^ (10).

The socio-demographic index (SDI), a composite measure reflecting regional economic development, was used to categorize regions. SDI integrates per capita income and educational attainment, with scores ranging from 0 to 1. Regions were classified into five groups based on SDI values: low (< 0.46), low-middle (0.46–0.60), middle (0.61–0.69), high-middle (0.70–0.81), and high (> 0.81). Additionally, projections of ASDR and age-standardized incidence rate (ASIR) for lymphoma and leukemia were estimated for the period 2022–2046 across different age cohorts.

### Disease prediction

To account for variations in population age structures, ASDR and ASMR were used to evaluate disease patterns of non-Hodgkin lymphoma, acute myeloid leukemia, and acute lymphoid leukemia across different regions. Compared to alternative methods such as generalized additive models and Poisson regression analyses, the Bayesian age-period-cohort (BAPC) framework demonstrated superior accuracy in predicting cancer-related statistics. In this study, the BAPC model was employed to project cancer-related ASMR and ASDR.

## Results

### Incidence and death of lymphoma and leukemia attributable to BMI worldwide

**Figure 1:**
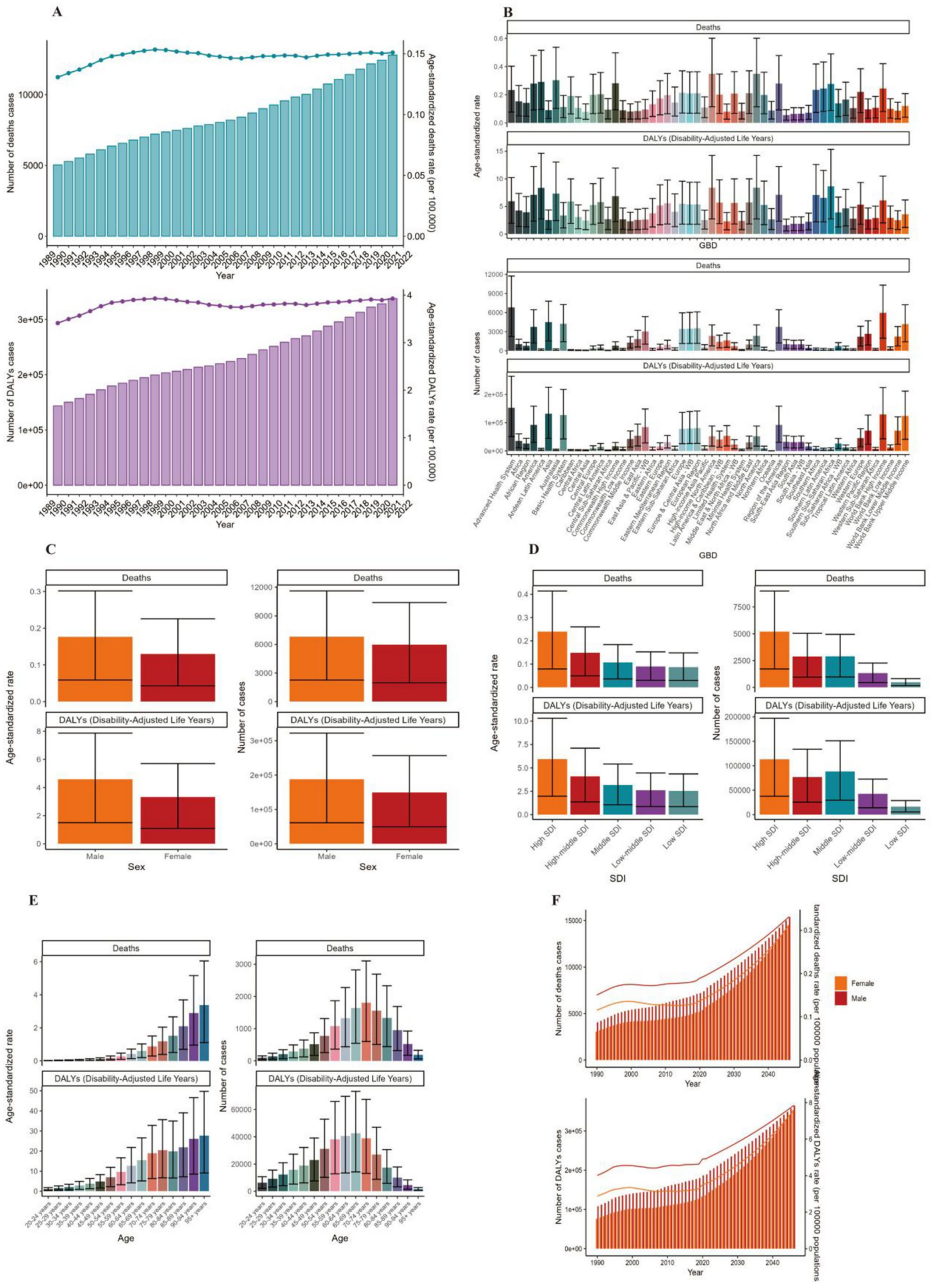
ASMR and ASDR of NHL. **(A)** Numbers and age-standardized rates of obesity-related deaths and DALYs from 1990 to 2021. **(B–E)** Numbers and age-standardized rates of obesity-related deaths and DALYs in 2021 for different region **(B)**, sex **(C)**, SDI region **(D)** and different age groups **(E)** in 2021. **(F)** The predicted results in obesity-related numbers and age-standardized rates of deaths and DALYs by sex globally from 1990 to 2046 of the BAPC model. ASMR, age-standardized mortality rate; ASDR, age-standardized DALYs rate; BAPC, Bayesian age-period-cohort.

**Figure 2:**
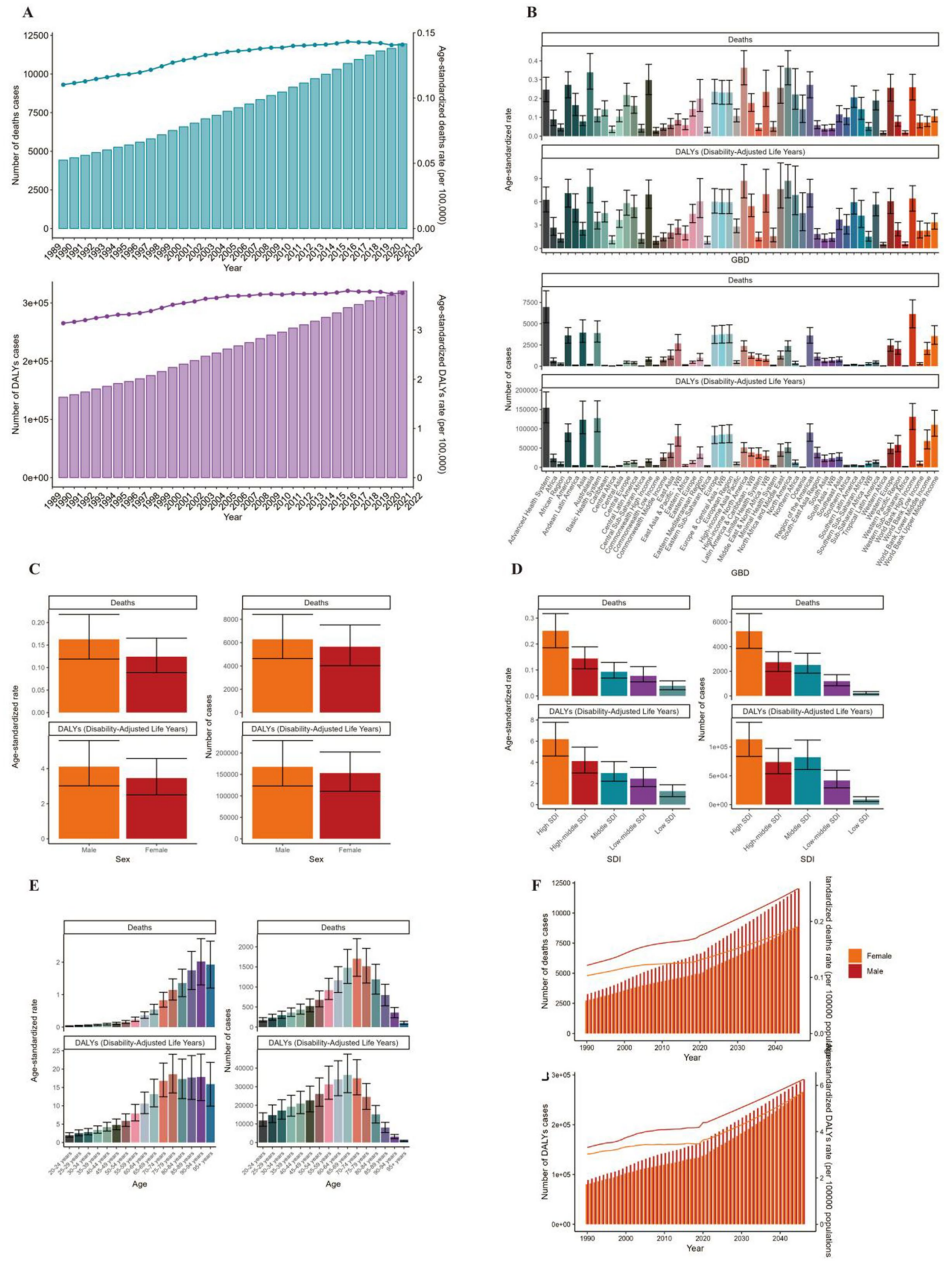
ASMR and ASDR of AML. **(A)** Numbers and age-standardized rates of obesity-related deaths and DALYs from 1990 to 2021. **(B–E)** Numbers and age-standardized rates of obesity-related deaths and DALYs in 2021 for different region **(B)**, sex **(C)**, SDI region **(D)** and different age groups **(E)** in 2021. **(F)** The predicted results in obesity-related numbers and age-standardized rates of deaths and DALYs by sex globally from 1990 to 2046 of the BAPC model. ASMR, age-standardized mortality rate; ASDR, age-standardized DALYs rate; BAPC, Bayesian age-period-cohort.

**Figure 3:**
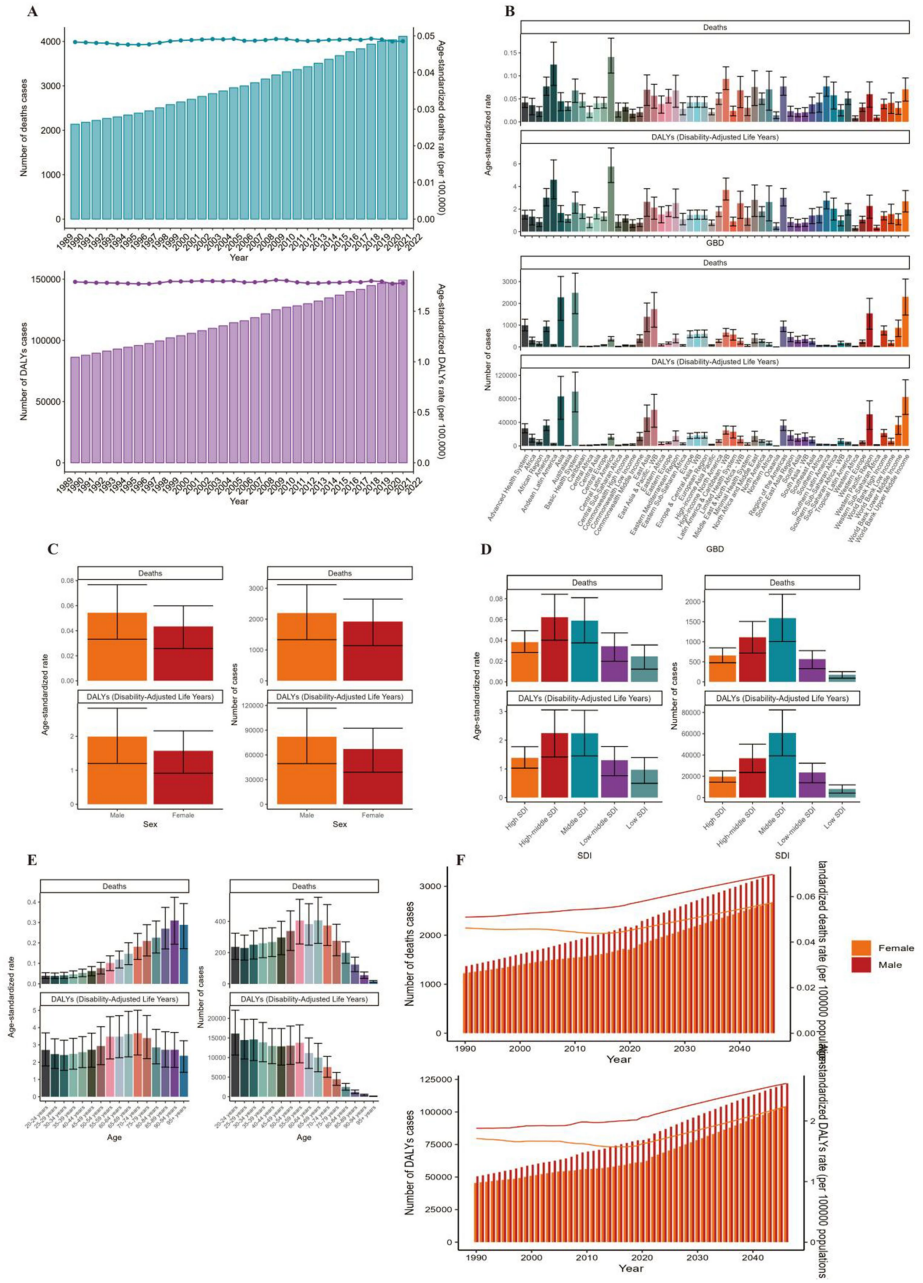
ASMR and ASDR of ALL. **(A)** Numbers and age-standardized rates of obesity-related deaths and DALYs from 1990 to 2021. **(B–E)** Numbers and age-standardized rates of obesity-related deaths and DALYs in 2021 for different region **(B)**, sex **(C)**, SDI region **(D)** and different age groups **(E)** in 2021. **(F)** The predicted results in obesity-related numbers and age-standardized rates of deaths and DALYs by sex globally from 1990 to 2046 of the BAPC model. ASMR, age-standardized mortality rate; ASDR, age-standardized DALYs rate; BAPC, Bayesian age-period-cohort.

Globally, the number of deaths cases and DALYs case of non-Hodgkin lymphoma, acute myeloid leukemia and acute lymphoid leukemia increased gradually since 1990 (Figures 1A, 2A, 3A; Supplementary Tables 1–3). The death cases of non-Hodgkin lymphoma attributable to BMI increased from 5,038 (95% UI: 1710–8,526) in 1990 to 12,796 (95% UI: 4253–21,966) in 2021 (Figure 1A; Table 1). For acute myeloid leukemia and acute lymphoid leukemia, the death cases rose from 4,426 cases (95% UI: 3,200–5,844) and 2,137 cases (95% UI: 1531–2,899) in 1990 to 11,947 cases (95% UI: 8,806–15,513) and 4,116 cases (95% UI: 2686–5,529) in 2021, respectively (Figures 2A, 3A; Tables 1, 2).

**Table 1:** The number of deaths cases and the age-standardized deaths rate attributable to high BMI in 1990 and 2021, and its trends from 1990 to 2021 globally in NHL.

Characteristics	1990		2021		1990–2021
	Number of deaths cases (95% UI)	The age-standardized deaths rate/100000 (95% UI)	Number of deaths cases (95% UI)	The age-standardized deaths rate/100000 (95% UI)	EAPC (95% CI)
Global	5,038 (1710–8,526)	0.13 (0.04–0.22)	12,796 (4253–21,966)	0.15 (0.05–0.26)	0.2 (0.08–0.32)
Sex					
Female	2,390 (808–4,084)	0.11 (0.04–0.2)	5,977 (1975–10,396)	0.13 (0.04–0.23)	0.05 (−0.1–0.19)
Male	2,648 (902–4,442)	0.15 (0.05–0.25)	6,819 (2259–11,614)	0.18 (0.06–0.3)	0.37 (0.27–0.47)
Age					
20–24 years	57 (20–94)	0.01 (0–0.02)	92 (31–155)	0.02 (0.01–0.03)	0.68 (0.59–0.76)
25–29 years	82 (28–135)	0.02 (0.01–0.03)	143 (47–240)	0.02 (0.01–0.04)	0.78 (0.73–0.82)
30–34 years	107 (37–180)	0.03 (0.01–0.05)	205 (69–347)	0.03 (0.01–0.06)	0.47 (0.34–0.6)
35–39 years	150 (51–252)	0.04 (0.01–0.07)	287 (95–496)	0.05 (0.02–0.09)	0.28 (0.1–0.46)
40–44 years	190 (65–325)	0.07 (0.02–0.11)	379 (125–647)	0.08 (0.02–0.13)	0.05 (−0.09–0.2)
45–49 years	237 (81–402)	0.1 (0.03–0.17)	514 (169–870)	0.11 (0.04–0.18)	−0.11 (−0.21–0.01)
50–54 years	353 (120–593)	0.17 (0.06–0.28)	776 (255–1,312)	0.17 (0.06–0.29)	−0.17 (−0.3–0.04)
55–59 years	474 (161–799)	0.26 (0.09–0.43)	1,081 (362–1869)	0.27 (0.09–0.47)	0.01 (−0.12–0.14)
60–64 years	617 (209–1,044)	0.38 (0.13–0.65)	1,326 (438–2,279)	0.41 (0.14–0.71)	0.04 (−0.04–0.11)
65–69 years	708 (237–1,202)	0.57 (0.19–0.97)	1,643 (547–2,824)	0.6 (0.2–1.02)	−0.06 (−0.15–0.03)
70–74 years	654 (221–1,105)	0.77 (0.26–1.3)	1802 (603–3,103)	0.88 (0.29–1.51)	−0.02 (−0.17–0.14)
75–79 years	640 (215–1,097)	1.04 (0.35–1.78)	1,559 (509–2,693)	1.18 (0.39–2.04)	0.05 (−0.13–0.23)
80–84 years	433 (147–743)	1.22 (0.42–2.1)	1,329 (441–2,334)	1.52 (0.5–2.66)	0.36 (0.15–0.56)
85–89 years	229 (79–393)	1.52 (0.52–2.6)	958 (318–1,689)	2.1 (0.7–3.69)	0.92 (0.69–1.15)
90–94 years	83 (28–146)	1.94 (0.66–3.41)	519 (171–924)	2.9 (0.95–5.17)	1.27 (1.11–1.43)
95 + years	22 (7–40)	2.18 (0.72–3.88)	184 (60–330)	3.38 (1.11–6.05)	1.24 (1.12–1.36)
SDI region					
High-middle SDI	1,137 (385–1917)	0.11 (0.04–0.19)	2,886 (957–5,051)	0.15 (0.05–0.26)	0.73 (0.64–0.83)
High SDI	2,738 (909–4,678)	0.25 (0.08–0.42)	5,188 (1722–8,990)	0.24 (0.08–0.41)	−0.54 (−0.72–0.36)
Low-middle SDI	304 (109–520)	0.05 (0.02–0.08)	1,333 (447–2,272)	0.09 (0.03–0.15)	2.06 (1.98–2.15)
Low SDI	136 (49–228)	0.06 (0.02–0.1)	482 (162–823)	0.09 (0.03–0.15)	1.23 (1.11–1.34)
Middle SDI	716 (261–1,195)	0.07 (0.02–0.11)	2,893 (973–4,948)	0.11 (0.04–0.18)	1.51 (1.46–1.57)

**Table 2:** The number of deaths cases and the age-standardized deaths rate attributable to high BMI in 1990 and 2021, and its trends from 1990 to 2021 globally in AML.

Characteristics	1990		2021		1990–2021
	Number of deaths cases (95% UI)	The age-standardized deaths rate/100000 (95% UI)	Number of deaths cases (95% UI)	The age-standardized deaths rate/100000 (95% UI)	EAPC (95% CI)
Global	4,426 (3200–5,844)	0.11 (0.08–0.15)	11,947 (8806–15,513)	0.14 (0.1–0.18)	0.87 (0.76–0.98)
Sex					
Female	2,233 (1600–3,012)	0.1 (0.07–0.14)	5,659 (4024–7,520)	0.12 (0.09–0.17)	0.64 (0.54–0.74)
Male	2,194 (1595–2,937)	0.12 (0.09–0.16)	6,288 (4636–8,421)	0.16 (0.12–0.22)	1.08 (0.96–1.2)
Age					
20–24 years	126 (85–183)	0.03 (0.02–0.04)	175 (128–235)	0.03 (0.02–0.04)	0.36 (0.27–0.45)
25–29 years	149 (104–207)	0.03 (0.02–0.05)	234 (170–320)	0.04 (0.03–0.05)	0.62 (0.52–0.72)
30–34 years	164 (116–226)	0.04 (0.03–0.06)	296 (216–396)	0.05 (0.04–0.07)	0.55 (0.47–0.63)
35–39 years	203 (144–277)	0.06 (0.04–0.08)	362 (266–477)	0.06 (0.05–0.09)	0.28 (0.23–0.34)
40–44 years	228 (164–304)	0.08 (0.06–0.11)	436 (322–572)	0.09 (0.06–0.11)	0.13 (0.04–0.21)
45–49 years	239 (173–321)	0.1 (0.07–0.14)	524 (380–701)	0.11 (0.08–0.15)	0.12 (0.01–0.23)
50–54 years	313 (226–414)	0.15 (0.11–0.19)	678 (504–902)	0.15 (0.11–0.2)	0.08 (−0.03–0.2)
55–59 years	396 (294–522)	0.21 (0.16–0.28)	924 (686–1,215)	0.23 (0.17–0.31)	0.35 (0.25–0.45)
60–64 years	506 (367–664)	0.32 (0.23–0.41)	1,165 (862–1,505)	0.36 (0.27–0.47)	0.5 (0.4–0.6)
65–69 years	567 (413–740)	0.46 (0.33–0.6)	1,477 (1087–1936)	0.54 (0.39–0.7)	0.61 (0.5–0.73)
70–74 years	513 (377–670)	0.61 (0.45–0.79)	1706 (1266–2,204)	0.83 (0.61–1.07)	0.97 (0.85–1.09)
75–79 years	492 (360–645)	0.8 (0.58–1.05)	1,515 (1097–1960)	1.15 (0.83–1.49)	1.28 (1.13–1.43)
80–84 years	309 (221–404)	0.87 (0.62–1.14)	1,190 (821–1,565)	1.36 (0.94–1.79)	1.61 (1.42–1.81)
85–89 years	159 (109–214)	1.05 (0.72–1.41)	800 (529–1,066)	1.75 (1.16–2.33)	2 (1.78–2.21)
90–94 years	50 (32–67)	1.16 (0.75–1.57)	362 (232–488)	2.02 (1.3–2.73)	2.16 (2.01–2.32)
95 + years	11 (7–16)	1.12 (0.69–1.54)	105 (66–144)	1.93 (1.2–2.65)	1.98 (1.89–2.07)
SDI region					
High-middle SDI	1,131 (825–1,493)	0.11 (0.08–0.15)	2,738 (1989–3,588)	0.14 (0.1–0.19)	0.89 (0.8–0.98)
High SDI	2,171 (1604–2,788)	0.2 (0.15–0.25)	5,253 (3862–6,682)	0.25 (0.19–0.32)	0.89 (0.74–1.05)
Low-middle SDI	297 (192–475)	0.04 (0.03–0.07)	1,195 (828–1728)	0.08 (0.05–0.11)	2.11 (2.02–2.19)
Low SDI	74 (37–126)	0.03 (0.01–0.05)	239 (142–354)	0.04 (0.02–0.06)	0.99 (0.9–1.08)
Middle SDI	747 (513–1,112)	0.06 (0.04–0.09)	2,509 (1847–3,466)	0.09 (0.07–0.13)	1.19 (1.13–1.26)

**Figure 4:**
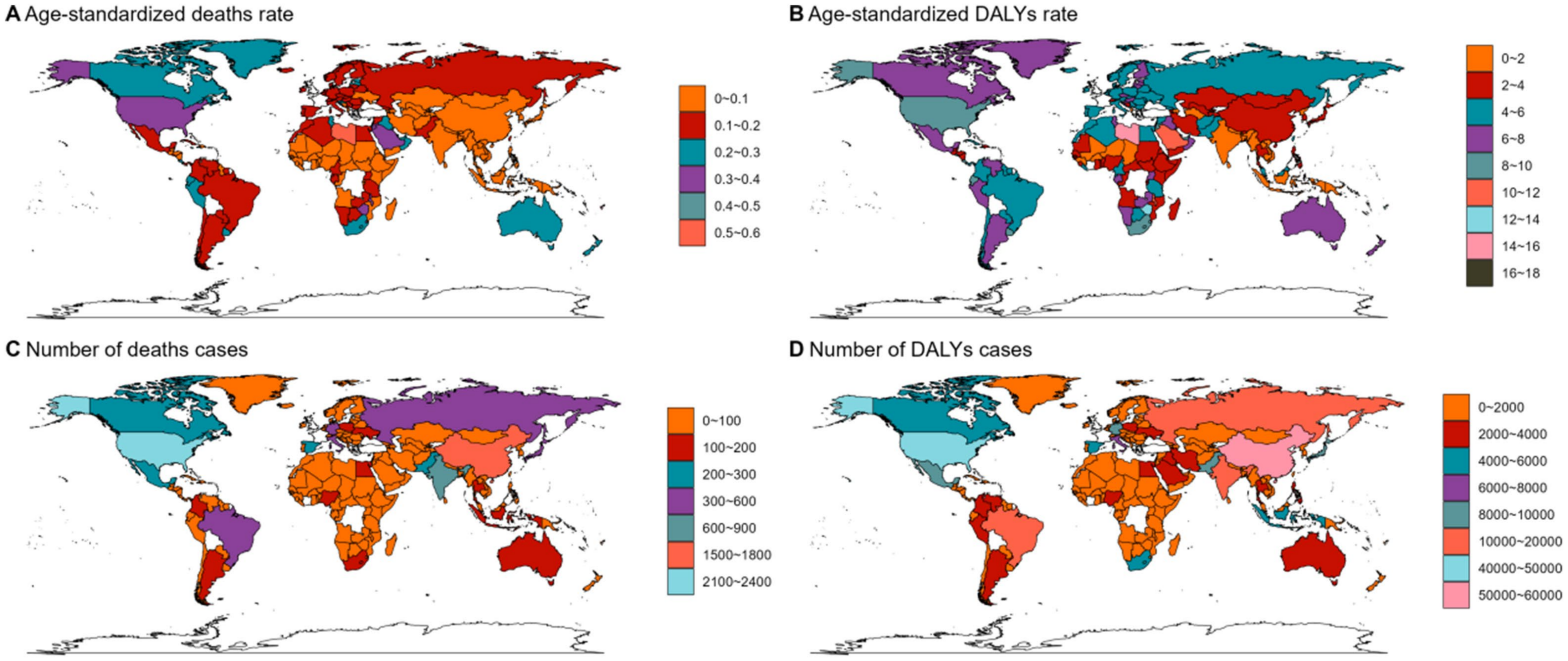
The ASMR **(A)**, ASDR **(B)**, number of deaths cases **(C)** and DALYs cases **(D)** for NHL in different regions and countries around the world. ASMR: Age-Standardized Mortality Rate, ASDR: Age-Standardized DALYs Rate.

**Figure 5:**
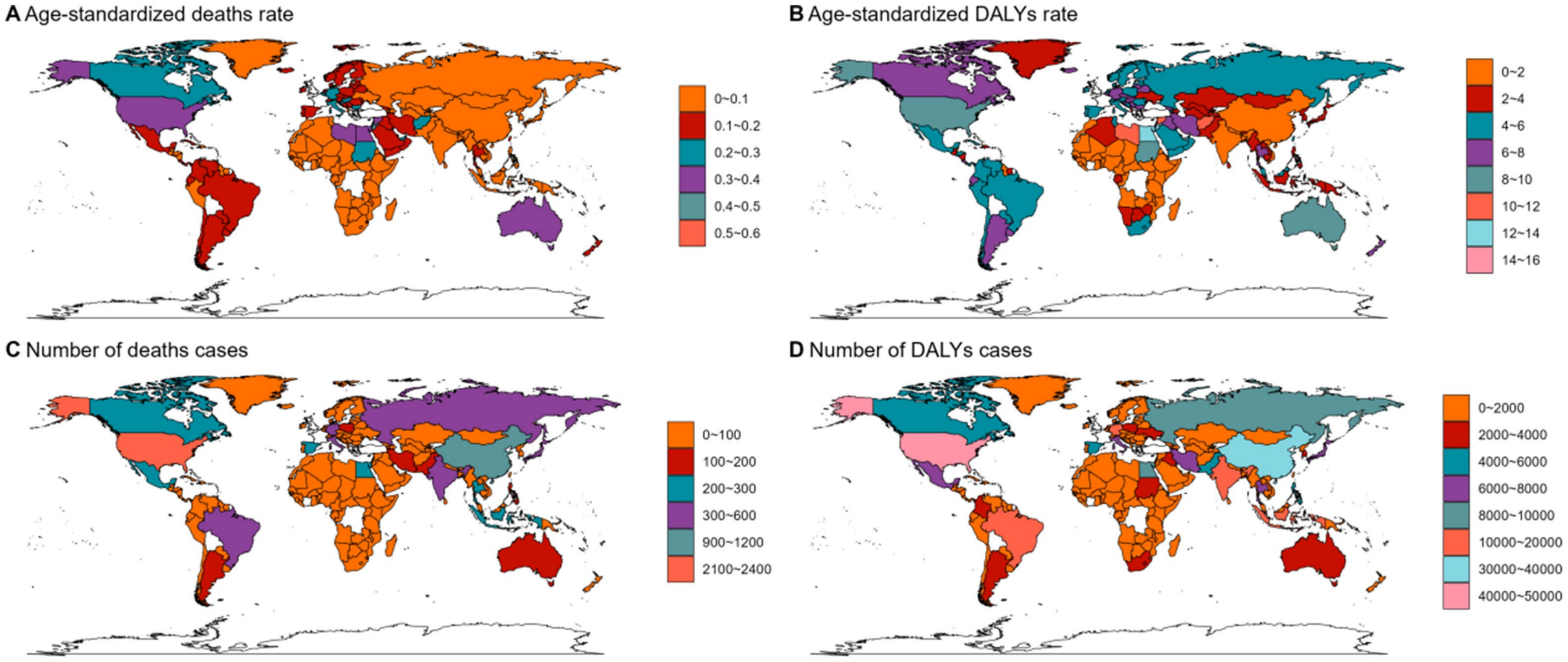
The ASMR **(A)**, ASDR **(B)**, number of deaths cases **(C)** and DALYs cases **(D)** for AML in different regions and countries around the world. ASMR: Age-Standardized Mortality Rate, ASDR: Age-Standardized DALYs Rate.

**Figure 6:**
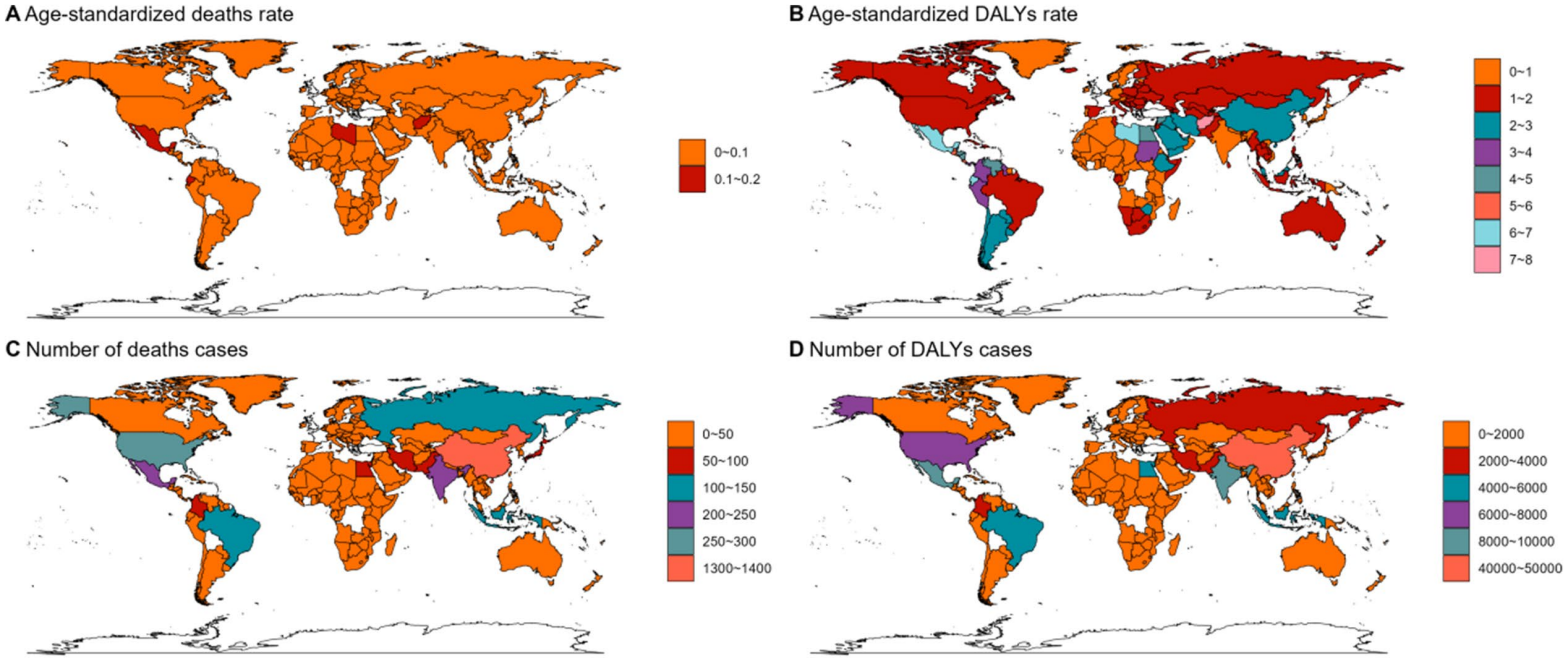
The ASMR **(A)**, ASDR **(B)**, number of deaths cases **(C)** and DALYs cases **(D)** for ALL in different regions and countries around the world. ASMR: Age-Standardized Mortality Rate, ASDR: Age-Standardized DALYs Rate.

In 2021, Asia ranked the top one in high BMI-related deaths (4,517, 95% UI 1,550–7,789) or DALYs (131,486, 95% UI: 44646–225,735) among the 54 GBD regions for non-Hodgkin, and Oceania ranked the bottom one for deaths (7, 95% UI: 2–13) and DALYs (267, 82–468) (Figures 1B, 2B, 3B, 4; Supplementary Table 1). The number of deaths cases attributable to high BMI was highest in Asia for acute myeloid leukemia (*n* = 3,942, 95% UI: 2804–5,455) and acute lymphoid leukemia (*n* = 2,280, 95% UI: 1220–3,235) (Figures 5, 6).

In 2021, the number of deaths cases and DALYs case were generally higher in male population than female population, while the relative rate was less than 1.5 (Figures 1C, 2C, 3C; Tables 1–3; Supplementary Tables 1–3).

**Table 3:** The number of deaths cases and the age-standardized deaths rate attributable to high BMI in 1990 and 2021, and its trends from 1990 to 2021 globally in ALL.

Characteristics	1990		2021		1990–2021
	Number of deaths cases (95% UI)	The age-standardized deaths rate/100000 (95% UI)	Number of deaths cases (95% UI)	The age-standardized deaths rate/100000 (95% UI)	EAPC (95% CI)
Global	2,137 (1531–2,899)	0.05 (0.03–0.07)	4,116 (2686–5,529)	0.05 (0.03–0.07)	0.07 (0.04–0.1)
Sex					
Female	1,057 (711–1,480)	0.05 (0.03–0.06)	1920 (1140–2,650)	0.04 (0.03–0.06)	−0.2 (−0.24–0.16)
Male	1,080 (712–1,522)	0.05 (0.03–0.07)	2,197 (1334–3,115)	0.05 (0.03–0.08)	0.3 (0.26–0.33)
Age					
20–24 years	201 (139–282)	0.04 (0.03–0.06)	237 (156–324)	0.04 (0.03–0.05)	−0.08 (−0.14–0.02)
25–29 years	170 (121–235)	0.04 (0.03–0.05)	229 (152–312)	0.04 (0.03–0.05)	0.09 (−0.01–0.19)
30–34 years	155 (109–215)	0.04 (0.03–0.06)	251 (159–340)	0.04 (0.03–0.06)	0.04 (−0.05–0.13)
35–39 years	172 (118–237)	0.05 (0.03–0.07)	260 (167–355)	0.05 (0.03–0.06)	−0.22 (−0.33–0.1)
40–44 years	163 (113–224)	0.06 (0.04–0.08)	268 (175–359)	0.05 (0.03–0.07)	−0.23 (−0.32–0.14)
45–49 years	148 (103–204)	0.06 (0.04–0.09)	297 (186–401)	0.06 (0.04–0.08)	0.03 (−0.06–0.11)
50–54 years	167 (116–231)	0.08 (0.05–0.11)	338 (214–467)	0.08 (0.05–0.11)	0 (−0.13–0.12)
55–59 years	191 (135–258)	0.1 (0.07–0.14)	406 (255–541)	0.1 (0.06–0.14)	−0.01 (−0.09–0.07)
60–64 years	197 (141–265)	0.12 (0.09–0.16)	382 (248–513)	0.12 (0.08–0.16)	0.11 (0.04–0.17)
65–69 years	178 (126–239)	0.14 (0.1–0.19)	406 (259–554)	0.15 (0.09–0.2)	0.09 (0.04–0.14)
70–74 years	147 (105–199)	0.17 (0.12–0.23)	373 (245–508)	0.18 (0.12–0.25)	0.09 (0.03–0.16)
75–79 years	125 (90–166)	0.2 (0.15–0.27)	276 (180–382)	0.21 (0.14–0.29)	0.23 (0.15–0.32)
80–84 years	73 (51–97)	0.21 (0.15–0.27)	198 (132–269)	0.23 (0.15–0.31)	0.48 (0.41–0.56)
85–89 years	36 (24–49)	0.24 (0.16–0.32)	124 (78–171)	0.27 (0.17–0.37)	0.63 (0.52–0.73)
90–94 years	12 (8–16)	0.27 (0.18–0.37)	55 (35–76)	0.31 (0.19–0.42)	0.61 (0.52–0.7)
95 + years	2 (2–3)	0.24 (0.15–0.34)	16 (9–21)	0.29 (0.17–0.39)	0.62 (0.5–0.74)
SDI region					
High-middle SDI	699 (502–940)	0.07 (0.05–0.09)	1,115 (719–1,507)	0.06 (0.04–0.08)	−0.2 (−0.26–0.15)
High SDI	493 (366–633)	0.05 (0.03–0.06)	658 (478–850)	0.04 (0.03–0.05)	−0.58 (−0.65–0.51)
Low-middle SDI	185 (107–269)	0.02 (0.01–0.03)	570 (330–779)	0.03 (0.02–0.05)	1.37 (1.28–1.46)
Low SDI	69 (36–124)	0.02 (0.01–0.04)	177 (91–255)	0.02 (0.01–0.04)	0 (−0.1–0.09)
Middle SDI	689 (467–980)	0.05 (0.04–0.07)	1,593 (1007–2,188)	0.06 (0.04–0.08)	0.46 (0.43–0.5)

At the SDI region level, the deaths cases were highest in the middle SDI region group in acute lymphoid leukemia, with 146,882 and DALYs cases at 2,684,589 (Figures 3D, 6; Table 3). The trend was significantly different for non-Hodgkin lymphoma and acute myeloid leukemia, with the highest age-standardized deaths rate and DALYs rate in the high SDI region (Figures 1D, 2D, 4, 5; Tables 1, 2).

The ASMR of non-Hodgkin lymphoma, acute myeloid leukemia and acute lymphoid leukemia attributable to high BMI grew from 0.13/100,000 (95% UI: 0.04/100,000–0.22/100,000), 0.11/100,000 (95% UI: 0.08/100,000–0.15/100,000) and 0.05/100,000 (95% UI: 0.03/100,000–0.07/100,000) in 1990 to 0.15/100,000 (95% UI: 0.05/100,000–0.26/100,000), 0.14/100,000 (95% UI: 0.1/100,000–0.18/100,000) and 0.05/100,000 (95% UI: 0.03/100,000–0.07/100,000) in 2021 with EAPCs as 0.2% (95% CI: 0.08–0.32) and 0.87% (95% CI: 0.76–0.98), 0.07% (95% CI: 0.04–0.1), respectively (Tables 1–3).

### Disease burden caused by lymphoma and acute leukemia attributable to BMI

The standardized DALY rate of non-Hodgkin lymphoma and acute myeloid leukemia attributable to high BMI elevated from 3.41/100,000 (95% UI: 1.16/100,000–5.78/100,000) and 3.14/100,000 (95% UI: 2.27/100,000–4.17/100,000) in 1990 to 3.93/100,000 (95% UI: 1.31/100,000–6.76/100,000) and 3.76/100,000 (95% UI: 2.77/100,000–4.87/100,000) in 2021, respectively (Figures 1A, 2A; Supplementary Tables 1, 2). However, the standardized DALY rate of acute lymphoid leukemia attributable to BMI decreased slightly from 1.79/100,000 (95% UI: 1.27/100,000–2.44/100,000) in 1990 to 1.78/100,000 (95% UI: 1.16/100,000–2.39/100,000) in 2021 (Figure 3A; Supplementary Table 3).

The age standard rate of Deaths and DALYs attributable to BMI in different age groups in 2021 were present in Figures 1E, 2E, 3E. For non-Hodgkin lymphoma, the age-standardized deaths rate increased with age and reached the top in the 95 + years old group. For acute myeloid leukemia and acute lymphoid leukemia, the DALYs rate increases with age and reaches the peak in the 70–79 age group, and then decreased (Figure 1E). However, the peak deaths rate appeared in the 90–94 age group (Figures 1E, 2E, 3E).

### Prediction of incidence and mortality of lymphoma and acute leukemia attributable to BMI

The BP neural network model with fine goodness of fit was selected to predict the incidence and mortality trend of lymphoma and leukemia attributable to BMI from 2022 to 2046. It was forecasted that the incidence and mortality status of lymphoma and acute leukemia attributable to BMI would continue to rise in the next 25-year cycle. The number of morbidity cases of non-Hodgkin lymphoma was likely to reach 15,403 and 14,490 for male and female in 2,046, respectively (Figure 1F). For acute myeloid leukemia and acute lymphoid leukemia, the number of morbidity cases in male population was likely to reach 12,025 and 3,242, while the number was likely to reach 8,866 and 2,665 in female population, respectively (Figures 2F, 3F).

## Discussion

Globally, the incidence, mortality, and DALY of leukemia and lymphoma increased over the last three decades. Our analysis confirms that BMI-related leukemia and lymphoma burden rose sharply from 1990 to 2021, with projections indicating continued escalation over the next 25 years, which was consistent with previous studies (8, 11). These findings underscore the urgent need to address metabolic risk factors in public health strategies targeting leukemian and lymphoma prevention.

In addition to genetic and virus infection, the onset of lymphoma and leukemia is also closely related to metabolic risks, such as high BMI and alcohol consumption (5, 6). BMI was also associated with the clinical efficacy and survival outcome in patients with hematological malignancies (12, 13). High BMI has contributed to hematological malignancies burden worldwide in 2021 and significant differences were observed in different groups, including sexes, ages, GBD regions, SDI regions and countries. From 1990 to 2021, the number of deaths and DALYs cases caused by high BMI were still very severe which showed an increasing trend. Furthermore, our predicted results showed that the number of deaths and DALYs cases would still increase in the next 25 years.

There are several potential mechanisms which may contribute to the increased morbidity of lymphoma and leukemia. Firstly, high BMI represents a chronic inflammatory condition and leads to changes in the adipocytokines in the plasma, including growth factors and pro-inflammatory cytokines, which were involved in biological processes such as immune response. Moreover, high BMI was associated with the secretion of leptin and adiponectin, which could result in insulin resistance and activation of the downstream pathways, contributing to cancer development. Recent studies demonstrated that obesity could modify the tumor microenvironment and promote cancer development, including the immune cell components and PD-1 expression (14–16). A group from UK reported a population-based cohort of 5.8 million individuals and revealed that 5 kg/m2 increase in BMI was related to a 10% increase in Hodgkin’s lymphoma (9). As an important risk factor of various cancers, it is necessary to evaluate the association of high BMI and hematological oncology.

From 1990 to 2021, as the global economy has advanced, the proportion of obese people increased significantly at the last three decades and the uptrend high BMI-related age-standardized burden was observed (14). In addition, with the extension of aging, the absolute number of morbidity and mortality will be always at a high level in the next 25-year cycle. It is necessary to attach great importance to reduce the obesity proportion. First, it is necessary to arouse public concern about the hazard of obesity through popularization of science. In terms of high-risk and older population, especially those with a family history of lymphoma and leukemia, early screening can be taken into consideration for early diagnosis and early treatment.

Our study showed that the standardized DALY rate of non-Hodgkin lymphoma and acute myeloid leukemia attributable to high BMI elevated from 1990 to 2021, while the standardized DALY rate of acute lymphoid leukemia decreased slightly. The prognosis of acute leukemia has significantly improved in the recent years (17), with a 3-year relapse-free survival over 80%, which could prolong survival and reduce disability rate.

Older population was one of the most vulnerable groups for the majority of cancers. According to the latest published data, the incidence of leukemia and non-Hodgkin lymphoma was significantly higher in the age > 65 population, with the peak incidence in the 65–84 years group (1). Older population was at more risk from various cancers due to immunocompromised translations and bad health condition. Consistent with the above result, our study revealed that the age > 65 years old group was vulnerable to non-Hodgkin lymphoma and acute leukemia, indicating that more health protection measures of the older adults should be established.

In this study, we found that high BMI-related lymphoma and leukemia burden was higher in males. The gender difference existed because obesity and overweight were more prevalent in the male population (18). In addition, some risk factors associated with cancer development was closely related to male, such as alcohol drinking, smoking, hypertension, and dyslipidemia (19, 20). However, the gender difference in the disease burden attributable to high BMI remained unclear and needs further investigation.

There was evidence that age was an important risk factor for cancer, with the older population being the most vulnerable demographic (21). Previous studies have demonstrated that older adults are more vulnerable to lymphoma (22, 23). In general, these results were all consistence with our study, so we needed to strengthen the relevant health protection of the older adults.

The prediction model of non-Hodgkin lymphoma and acute leukemia revealed that the deaths and DALYs cases would increase significantly by different degrees from 2021 to 2046 in both male and female population. Previous studies also showed that obesity-related cancer burden would increase in the next two decades. This was possibly due to a combination of demographic changes, unhealthy lifestyle, stress obesity and socioeconomic development over the coming years. Therefore, it is necessary to take prevention measures to inhibit this trend for this disease.

This study has several limitations. Firstly, detailed data from different counties and provinces was missed and the regional difference may lead to bias of results. Secondly, some primary data in the GBD database was not available and the disease burden was estimated by a standardized Bayesian regression tool, DisMod-MR, which may not represent the real situation. Thirdly, the prediction model was built when other factors were assumed to be constant in the next 25 years and the predictive value was limited. Moreover, the GBD study’s macro-level perspective inherently limits its ability to fully resolve variations in subtypes, genetic profiles, and comorbidities within the highly heterogeneous domain of leukemia and lymphoma.

## Conclusion

Compared with traditional genetic factors, this study focuses on the non-genetic factor, BMI for the development of lymphoma and leukemia. More attention is paid to the disease burden and its development trend caused by lymphoma and leukemia attributable to BMI. It is revealed that both incidence and mortality are on the rise and the upward trend will continue over the next two decades worldwide. The above results indicated that high BMI remained a public health problem and needed to be addressed in the future. External intervention should be developed to constrain the development of high BMI-related leukemia and lymphoma.

## Data Availability

The original contributions presented in the study are included in the article/Supplementary material, further inquiries can be directed to the corresponding author.

## Footnotes

^1^ https://ghdx.healthdata.org
