# Supplementary material for: Global, regional, and national disease burden of lymphoma and leukemia attributable to high body mass index: from 1990 to 2021
Source: Front Nutr. 2025 Jul 24;12:1592443. doi: 10.3389/fnut.2025.1592443 (PMC12328193; doi:10.3389/fnut.2025.1592443)
Supplement: Supplementary file 1 [file Table_1.docx]

Supplementary table 1. The number of DALYs cases and the age-standardized DALYs rate attributable to obesity in 1990 and 2021, and its trends from 1990 to 2021 globally in NHL.

| Characteristics | 1990 | | 2021 | | 1990-2021 |
| --- | --- | --- | --- | --- | --- |
|  | Number of DALYs cases (95% UI) | The age-standardized DALYs rate/100000 (95% UI) | Number of DALYs cases (95% UI) | The age-standardized DALYs rate/100000 (95% UI) | EAPC (95% CI) |
| Global | 143572 (48858-242899) | 3.41 (1.16-5.78) | 337747 (112318-580879) | 3.93 (1.31-6.76) | 0.19 (0.08-0.29) |
| Sex |  |  |  |  |  |
| Female | 62715 (21260-106379) | 2.86 (0.97-4.86) | 149751 (49477-256730) | 3.33 (1.1-5.71) | 0.12 (-0.01-0.25) |
| Male | 80856 (27597-134805) | 4 (1.36-6.68) | 187995 (61452-322062) | 4.59 (1.5-7.86) | 0.26 (0.17-0.35) |
| Age |  |  |  |  |  |
| 20-24 years | 3944 (1390-6477) | 0.8 (0.28-1.32) | 6363 (2163-10718) | 1.07 (0.36-1.79) | 0.7 (0.61-0.79) |
| 25-29 years | 5287 (1822-8739) | 1.19 (0.41-1.97) | 9277 (3045-15558) | 1.58 (0.52-2.64) | 0.8 (0.76-0.84) |
| 30-34 years | 6398 (2219-10775) | 1.66 (0.58-2.8) | 12349 (4122-20900) | 2.04 (0.68-3.46) | 0.49 (0.36-0.63) |
| 35-39 years | 8164 (2762-13727) | 2.32 (0.78-3.9) | 15796 (5263-27259) | 2.82 (0.94-4.86) | 0.3 (0.12-0.48) |
| 40-44 years | 9381 (3208-15985) | 3.27 (1.12-5.58) | 18808 (6186-32144) | 3.76 (1.24-6.43) | 0.08 (-0.07-0.22) |
| 45-49 years | 10505 (3574-17838) | 4.52 (1.54-7.68) | 23070 (7571-39138) | 4.87 (1.6-8.27) | -0.08 (-0.18-0.02) |
| 50-54 years | 13907 (4722-23378) | 6.54 (2.22-11) | 31118 (10196-52970) | 6.99 (2.29-11.91) | -0.12 (-0.26-0.02) |
| 55-59 years | 16428 (5565-27793) | 8.87 (3-15.01) | 38228 (12843-65942) | 9.66 (3.25-16.66) | 0.08 (-0.06-0.21) |
| 60-64 years | 18467 (6243-31308) | 11.5 (3.89-19.49) | 40638 (13447-69764) | 12.7 (4.2-21.8) | 0.12 (0.05-0.2) |
| 65-69 years | 17906 (6006-30498) | 14.49 (4.86-24.67) | 42594 (14198-73393) | 15.44 (5.15-26.61) | 0.03 (-0.06-0.12) |
| 70-74 years | 13699 (4630-23225) | 16.18 (5.47-27.43) | 39001 (13067-67502) | 18.95 (6.35-32.79) | 0.09 (-0.06-0.25) |
| 75-79 years | 10683 (3581-18346) | 17.35 (5.82-29.8) | 26962 (8828-47031) | 20.44 (6.69-35.66) | 0.16 (-0.03-0.34) |
| 80-84 years | 5544 (1887-9535) | 15.67 (5.33-26.95) | 17358 (5767-30620) | 19.82 (6.58-34.96) | 0.43 (0.22-0.63) |
| 85-89 years | 2339 (804-4028) | 15.48 (5.32-26.66) | 10010 (3298-17853) | 21.89 (7.21-39.05) | 1 (0.77-1.23) |
| 90-94 years | 735 (250-1290) | 17.15 (5.84-30.11) | 4661 (1525-8336) | 26.06 (8.52-46.6) | 1.33 (1.17-1.49) |
| 95+ years | 183 (61-326) | 18 (5.95-32.07) | 1513 (496-2711) | 27.75 (9.09-49.74) | 1.21 (1.08-1.33) |
| SDI region |  |  |  |  |  |
| High-middle SDI | 33754 (11449-56964) | 3.24 (1.1-5.46) | 76779 (25512-133880) | 4.09 (1.36-7.11) | 0.63 (0.55-0.7) |
| High SDI | 70457 (23344-120429) | 6.6 (2.19-11.28) | 113176 (37621-197050) | 5.94 (1.98-10.3) | -0.78 (-0.97--0.6) |
| Low-middle SDI | 10209 (3652-17412) | 1.38 (0.49-2.36) | 42549 (14147-72671) | 2.61 (0.87-4.45) | 2.05 (1.96-2.14) |
| Low SDI | 4551 (1621-7638) | 1.65 (0.59-2.75) | 16596 (5542-28697) | 2.54 (0.85-4.36) | 1.22 (1.11-1.32) |
| Middle SDI | 24415 (8869-40779) | 1.94 (0.7-3.24) | 88276 (29589-150986) | 3.17 (1.06-5.42) | 1.5 (1.45-1.55) |
