# Supplementary material for: Global, regional, and national disease burden of lymphoma and leukemia attributable to high body mass index: from 1990 to 2021
Source: Front Nutr. 2025 Jul 24;12:1592443. doi: 10.3389/fnut.2025.1592443 (PMC12328193; doi:10.3389/fnut.2025.1592443)
Supplement: Supplementary file 2 [file Table_2.docx]

Supplementary table 2. The number of DALYs cases and the age-standardized DALYs rate attributable to obesity in 1990 and 2021, and its trends from 1990 to 2021 globally in AML.

| Characteristics | 1990 | | 2021 | | 1990-2021 |
| --- | --- | --- | --- | --- | --- |
|  | Number of DALYs cases (95% UI) | The age-standardized DALYs rate/100000 (95% UI) | Number of DALYs cases (95% UI) | The age-standardized DALYs rate/100000 (95% UI) | EAPC (95% CI) |
| Global | 138174 (99984-184639) | 3.14 (2.27-4.17) | 320774 (236255-415689) | 3.76 (2.77-4.87) | 0.61 (0.52-0.7) |
| Sex |  |  |  |  |  |
| Female | 68961 (49292-94392) | 3.03 (2.17-4.13) | 153153 (110712-202175) | 3.47 (2.5-4.58) | 0.43 (0.35-0.51) |
| Male | 69213 (50212-93967) | 3.3 (2.39-4.46) | 167621 (123010-228686) | 4.11 (3.02-5.6) | 0.78 (0.67-0.88) |
| Age |  |  |  |  |  |
| 20-24 years | 8597 (5814-12421) | 1.75 (1.18-2.52) | 11865 (8724-15963) | 1.99 (1.46-2.67) | 0.36 (0.27-0.45) |
| 25-29 years | 9431 (6597-13082) | 2.13 (1.49-2.96) | 14739 (10719-20211) | 2.51 (1.82-3.44) | 0.62 (0.52-0.72) |
| 30-34 years | 9552 (6754-13098) | 2.48 (1.75-3.4) | 17185 (12545-23024) | 2.84 (2.08-3.81) | 0.55 (0.48-0.63) |
| 35-39 years | 10789 (7672-14704) | 3.06 (2.18-4.17) | 19238 (14142-25392) | 3.43 (2.52-4.53) | 0.28 (0.23-0.34) |
| 40-44 years | 10972 (7930-14671) | 3.83 (2.77-5.12) | 20991 (15531-27548) | 4.2 (3.1-5.51) | 0.12 (0.04-0.2) |
| 45-49 years | 10337 (7483-13912) | 4.45 (3.22-5.99) | 22676 (16474-30330) | 4.79 (3.48-6.41) | 0.12 (0.01-0.23) |
| 50-54 years | 12031 (8718-15931) | 5.66 (4.1-7.49) | 26141 (19468-34736) | 5.88 (4.38-7.81) | 0.09 (-0.03-0.2) |
| 55-59 years | 13366 (9931-17628) | 7.22 (5.36-9.52) | 31261 (23255-41101) | 7.9 (5.88-10.39) | 0.36 (0.25-0.46) |
| 60-64 years | 14731 (10681-19360) | 9.17 (6.65-12.05) | 33981 (25170-43969) | 10.62 (7.86-13.74) | 0.51 (0.41-0.61) |
| 65-69 years | 13937 (10141-18185) | 11.28 (8.2-14.71) | 36346 (26795-47571) | 13.18 (9.71-17.25) | 0.62 (0.51-0.74) |
| 70-74 years | 10379 (7632-13590) | 12.26 (9.01-16.05) | 34536 (25642-44556) | 16.78 (12.46-21.65) | 0.97 (0.86-1.09) |
| 75-79 years | 7967 (5827-10394) | 12.94 (9.47-16.89) | 24527 (17797-31727) | 18.6 (13.49-24.06) | 1.28 (1.12-1.43) |
| 80-84 years | 3936 (2812-5145) | 11.13 (7.95-14.54) | 15142 (10473-19907) | 17.29 (11.96-22.73) | 1.6 (1.41-1.79) |
| 85-89 years | 1614 (1107-2164) | 10.68 (7.33-14.32) | 8089 (5364-10832) | 17.69 (11.73-23.69) | 1.98 (1.77-2.2) |
| 90-94 years | 439 (283-593) | 10.25 (6.6-13.83) | 3192 (2042-4311) | 17.84 (11.41-24.1) | 2.16 (2-2.32) |
| 95+ years | 95 (58-130) | 9.29 (5.7-12.79) | 865 (540-1190) | 15.88 (9.91-21.84) | 1.94 (1.85-2.03) |
| SDI region |  |  |  |  |  |
| High-middle SDI | 37302 (27276-49939) | 3.5 (2.56-4.67) | 73914 (53582-97392) | 4.12 (2.99-5.44) | 0.48 (0.41-0.55) |
| High SDI | 58021 (42968-74082) | 5.5 (4.08-7.04) | 113163 (83514-142499) | 6.2 (4.6-7.78) | 0.5 (0.35-0.65) |
| Low-middle SDI | 11225 (7118-17558) | 1.39 (0.9-2.21) | 41917 (29161-59660) | 2.46 (1.71-3.52) | 2.01 (1.93-2.09) |
| Low SDI | 2691 (1311-4668) | 0.9 (0.45-1.55) | 9159 (5467-13718) | 1.27 (0.76-1.88) | 1.04 (0.95-1.14) |
| Middle SDI | 28736 (19722-42705) | 2.1 (1.44-3.1) | 82229 (60811-111791) | 2.99 (2.21-4.07) | 1.07 (1-1.13) |
