# Supplementary material for: Global, regional, and national disease burden of lymphoma and leukemia attributable to high body mass index: from 1990 to 2021
Source: Front Nutr. 2025 Jul 24;12:1592443. doi: 10.3389/fnut.2025.1592443 (PMC12328193; doi:10.3389/fnut.2025.1592443)
Supplement: Supplementary file 3 [file Table_3.docx]

Supplementary table 3. The number of DALYs cases and the age-standardized DALYs rate attributable to obesity in 1990 and 2021, and its trends from 1990 to 2021 globally in ALL.

| Characteristics | 1990 | | 2021 | | 1990-2021 |
| --- | --- | --- | --- | --- | --- |
|  | Number of DALYs cases (95% UI) | The age-standardized DALYs rate/100000 (95% UI) | Number of DALYs cases (95% UI) | The age-standardized DALYs rate/100000 (95% UI) | EAPC (95% CI) |
| Global | 86249 (61159-117876) | 1.79 (1.27-2.44) | 149302 (97226-200414) | 1.78 (1.16-2.39) | 0.01 (-0.01-0.03) |
| Sex |  |  |  |  |  |
| Female | 41244 (27359-58762) | 1.71 (1.14-2.43) | 67211 (39013-92461) | 1.57 (0.91-2.16) | -0.29 (-0.32--0.25) |
| Male | 45005 (29484-62858) | 1.88 (1.23-2.63) | 82091 (49485-116713) | 1.99 (1.2-2.82) | 0.25 (0.22-0.28) |
| Age |  |  |  |  |  |
| 20-24 years | 13704 (9423-19168) | 2.78 (1.91-3.9) | 16142 (10640-22073) | 2.7 (1.78-3.7) | -0.08 (-0.14--0.02) |
| 25-29 years | 10717 (7639-14830) | 2.42 (1.73-3.35) | 14488 (9630-19722) | 2.46 (1.64-3.35) | 0.1 (-0.01-0.2) |
| 30-34 years | 8979 (6355-12502) | 2.33 (1.65-3.24) | 14614 (9227-19780) | 2.42 (1.53-3.27) | 0.05 (-0.04-0.14) |
| 35-39 years | 9126 (6284-12615) | 2.59 (1.78-3.58) | 13873 (8932-18957) | 2.47 (1.59-3.38) | -0.21 (-0.33--0.09) |
| 40-44 years | 7853 (5474-10827) | 2.74 (1.91-3.78) | 12942 (8442-17410) | 2.59 (1.69-3.48) | -0.23 (-0.32--0.14) |
| 45-49 years | 6426 (4439-8816) | 2.77 (1.91-3.8) | 12874 (8093-17412) | 2.72 (1.71-3.68) | 0.03 (-0.05-0.11) |
| 50-54 years | 6444 (4457-8897) | 3.03 (2.1-4.19) | 13064 (8260-18037) | 2.94 (1.86-4.05) | 0 (-0.12-0.13) |
| 55-59 years | 6448 (4552-8737) | 3.48 (2.46-4.72) | 13757 (8661-18345) | 3.48 (2.19-4.64) | 0 (-0.09-0.08) |
| 60-64 years | 5754 (4100-7733) | 3.58 (2.55-4.81) | 11149 (7226-15011) | 3.48 (2.26-4.69) | 0.11 (0.05-0.18) |
| 65-69 years | 4379 (3107-5892) | 3.54 (2.51-4.77) | 9996 (6368-13627) | 3.62 (2.31-4.94) | 0.09 (0.04-0.15) |
| 70-74 years | 2983 (2128-4033) | 3.52 (2.51-4.76) | 7566 (4972-10305) | 3.68 (2.42-5.01) | 0.09 (0.03-0.15) |
| 75-79 years | 2026 (1450-2699) | 3.29 (2.35-4.38) | 4479 (2907-6201) | 3.4 (2.2-4.7) | 0.23 (0.14-0.31) |
| 80-84 years | 922 (652-1222) | 2.6 (1.84-3.46) | 2502 (1666-3416) | 2.86 (1.9-3.9) | 0.47 (0.39-0.55) |
| 85-89 years | 366 (247-490) | 2.42 (1.64-3.24) | 1241 (783-1719) | 2.71 (1.71-3.76) | 0.62 (0.51-0.72) |
| 90-94 years | 103 (67-137) | 2.4 (1.57-3.21) | 486 (304-665) | 2.71 (1.7-3.72) | 0.61 (0.52-0.7) |
| 95+ years | 21 (13-28) | 2.02 (1.25-2.79) | 130 (77-177) | 2.38 (1.41-3.24) | 0.58 (0.45-0.7) |
| SDI region |  |  |  |  |  |
| High-middle SDI | 27538 (19707-37262) | 2.51 (1.8-3.4) | 37008 (23560-50188) | 2.25 (1.42-3.05) | -0.4 (-0.45--0.35) |
| High SDI | 17140 (12665-22033) | 1.71 (1.26-2.19) | 19621 (14530-25113) | 1.39 (1.03-1.77) | -0.59 (-0.66--0.52) |
| Low-middle SDI | 8200 (4818-12050) | 0.91 (0.53-1.33) | 23686 (13934-32317) | 1.3 (0.76-1.78) | 1.31 (1.22-1.4) |
| Low SDI | 3054 (1644-5393) | 0.89 (0.46-1.58) | 8198 (4294-11936) | 0.96 (0.5-1.4) | 0.03 (-0.07-0.13) |
| Middle SDI | 30234 (20367-42752) | 2.01 (1.36-2.86) | 60686 (39182-82326) | 2.24 (1.45-3.04) | 0.4 (0.36-0.43) |
